# *Anaplasma phagocytophilum* Infection Subverts Carbohydrate Metabolic Pathways in the Tick Vector, *Ixodes scapularis*

**DOI:** 10.3389/fcimb.2017.00023

**Published:** 2017-02-07

**Authors:** Alejandro Cabezas-Cruz, Pilar Alberdi, James J. Valdés, Margarita Villar, José de la Fuente

**Affiliations:** ^1^Institute of Parasitology, Biology Center, Czech Academy of SciencesCeské Budejovice, Czechia; ^2^Faculty of Science, University of South BohemiaCeské Budejovice, Czechia; ^3^SaBio. Instituto de Investigación en Recursos Cinegéticos (CSIC-UCLM-JCCM)Ciudad Real, Spain; ^4^Department of Virology, Veterinary Research InstituteBrno, Czechia; ^5^Department of Veterinary Pathobiology, Center for Veterinary Health Sciences, Oklahoma State UniversityStillwater, OK, USA

**Keywords:** proteomics, transcriptomics, glucose metabolism, *Ixodes scapularis*, *Anaplasma phagocytophilum*

## Abstract

The obligate intracellular pathogen, *Anaplasma phagocytophilum*, is the causative agent of human, equine, and canine granulocytic anaplasmosis and tick-borne fever (TBF) in ruminants. *A. phagocytophilum* has become an emerging tick-borne pathogen in the United States, Europe, Africa, and Asia, with increasing numbers of infected people and animals every year. It has been recognized that intracellular pathogens manipulate host cell metabolic pathways to increase infection and transmission in both vertebrate and invertebrate hosts. However, our current knowledge on how *A. phagocytophilum* affect these processes in the tick vector, *Ixodes scapularis* is limited. In this study, a genome-wide search for components of major carbohydrate metabolic pathways was performed in *I. scapularis* ticks for which the genome was recently published. The enzymes involved in the seven major carbohydrate metabolic pathways glycolysis, gluconeogenesis, pentose phosphate, tricarboxylic acid cycle (TCA), glyceroneogenesis, and mitochondrial oxidative phosphorylation and β-oxidation were identified. Then, the available transcriptomics and proteomics data was used to characterize the mRNA and protein levels of *I. scapularis* major carbohydrate metabolic pathway components in response to *A. phagocytophilum* infection of tick tissues and cultured cells. The results showed that major carbohydrate metabolic pathways are conserved in ticks. *A. phagocytophilum* infection inhibits gluconeogenesis and mitochondrial metabolism, but increases the expression of glycolytic genes. A model was proposed to explain how *A. phagocytophilum* could simultaneously control tick cell glucose metabolism and cytoskeleton organization, which may be achieved in part by up-regulating and stabilizing hypoxia inducible factor 1 alpha in a hypoxia-independent manner. The present work provides a more comprehensive view of the major carbohydrate metabolic pathways involved in the response to *A. phagocytophilum* infection in ticks, and provides the basis for further studies to develop novel strategies for the control of granulocytic anaplasmosis.

## Introduction

*Anaplasma phagocytophilum* (Rickettsiales: Anaplasmataceae) is an obligate intracellular bacterium mainly transmitted by *Ixodes* spp. ticks. This emerging pathogen has been reported in the United States, Europe, Africa, and Asia (de la Fuente et al., 2008; Stuen et al., 2013; Kocan et al., 2015), causing human granulocytic anaplasmosis (HGA), equine and canine granulocytic anaplasmosis and tick-borne fever (TBF) of ruminants (de la Fuente et al., 2008; Stuen et al., 2013; Kocan et al., 2015).

The development of *A. phagocytophilum* is complex and coordinated with the tick feeding cycle. Infection and multiplication in ticks occurs first in midgut cells during blood feeding, and then subsequently in other tissues including hemocytes and salivary glands from where transmission occurs to susceptible hosts (Kocan et al., 2015). To establish infection, *A. phagocytophilum* affect mechanisms that appear to be common to ticks and vertebrate hosts (de la Fuente et al., 2016a). These mechanisms include but are not limited to remodeling of the cytoskeleton, inhibition of cell apoptosis, manipulation of the immune response, and modification of cell epigenetics and metabolism (de la Fuente et al., 2016a).

Recently, transcriptomics, proteomics and metabolomics analyses of infected *I. scapularis* ISE6 cells showed that *A. phagocytophilum* infection affects glucose metabolic pathways (Villar et al., 2015). These results suggested that *A. phagocytophilum* manipulate carbohydrate metabolism to facilitate infection and multiplication in tick cells. However, the mechanisms used by *A. phagocytophilum* for the manipulation of carbohydrate metabolic pathways have not been fully characterized.

To better characterize the mechanisms used by *A. phagocytophilum* to manipulate carbohydrate metabolic pathways during infection of tick cells, the dynamics of the carbohydrate metabolism was characterized in the tick vector, *I. scapularis* in response to pathogen infection. First, the composition of major carbohydrate metabolic pathways was annotated using the recently published genome of *I. scapularis* (Gulia-Nuss et al., 2016). Then, previously published transcriptomics and proteomics data (Ayllón et al., 2015; Villar et al., 2015) was used to characterize the mRNA and protein levels of carbohydrate metabolic pathway components in response to *A. phagocytophilum* infection of *I. scapularis* nymphs, female midguts and salivary glands, and ISE6 cultured tick cells. Finally, functional studies were conducted in ISE6 tick cells to provide additional support for the role of these components during pathogen infection. These results expanded our knowledge of the different pathways affected by *A. phagocytophilum* infection in ticks, and provided new potential targets for the development of therapeutic and prevention strategies for the control of granulocytic anaplasmosis and other tick-borne diseases.

## Materials and methods

### Annotation of the major carbohydrate metabolic pathway components in the *I. scapularis* genome

The *I. scapularis* genome (Gulia-Nuss et al., 2016) was searched with the specific names of genes encoding for enzymes involved in the major carbohydrate metabolic pathways, glycolysis, gluconeogenesis, pentose phosphate, tricarboxylic acid cycle (TCA), glyceroneogenesis, and mitochondrial oxidative phosphorylation and β-oxidation. When records were not obtained using specific enzyme names, then the *I. scapularis* genome was searched with the Blastp tool from the Basic Local Alignment Search Tool (BLAST) using the human ortholog as “query” (Altschul et al., 1990; Madden et al., 1996). The sequences with the lowest *E*-value were selected. The conserved domains of identified protein sequences were classified using the protein families database Pfam (Finn et al., 2014). The *I. scapularis* orthologs found in the genome were double-checked by searching the *Homo sapiens* genome database using as queries the tick homologs identified in the previous step.

### Characterization of the *I. scapularis* mRNA and protein levels in response to *A. phagocytophilum* infection

The quantitative transcriptomics and proteomics data for uninfected and *A. phagocytophilum*-infected *I. scapularis* nymphs, female midguts and salivary glands, and ISE6 cultured cells were obtained from previously published results (Ayllón et al., 2015; Villar et al., 2015) and deposited at the Dryad repository database, NCBI's Gene Expression Omnibus database and ProteomeXchange Consortium via the PRIDE partner repository with the dataset identifier PXD002181 and doi: 10.6019/PXD002181. For transcriptomics and proteomics analysis in *I. scapularis* nymphs, female midguts and salivary glands, the procedures were described in Ayllón et al. (2015). Briefly, nymphs and adult female *I. scapularis* were infected with *A. phagocytophilum* by feeding on a sheep inoculated intravenously with ~1 × 10^7^ *A. phagocytophilum* (NY18 isolate)-infected HL-60 cells (90–100% infected cells). In this model, over 85% of ticks become infected with *A. phagocytophilum* in nymphs, midguts and salivary glands. Ticks (200 nymphs and 100 female adults) were removed from the sheep 7 days after infestation, held in the humidity chamber for 4 days and dissected for DNA, RNA, and protein extraction from whole internal tissues (nymphs) or midguts and salivary glands (adult females). Adult midguts and salivary glands were washed in PBS after collection to remove hemolymphs-related cells. Uninfected ticks were prepared in a similar way but feeding on an uninfected sheep. Two independent samples were collected and processed for each tick developmental stage and tissue. After RNA sequencing on an Illumina Hiseq 2000, TopHat was used to align the reads to the *I. scapularis* (assembly JCVI_ISG_i3_1.0; http://www.ncbi.nlm.nih.gov/nuccore/NZ_ABJB000000000) reference genome. Raw counts per gene were estimated by the Python script HTSeq count [http://www-huber.embl.de/users/anders/HTSeq/] using the reference genome. The raw counts per gene were used by DEGseq to estimate differential expression at *P* < 0.05. For peptide identification by liquid chromatography-tandem mass spectrometry (LC-MS/MS) using iTRAQ labeled peptides, all spectra were analyzed with Proteome Discoverer (version 1.4.0.29, Thermo Fisher Scientific) using a Uniprot database (http://www.uniprot.org) containing all sequences from Ixodida, Anaplasmataceae and Ruminantia. Peptide identification was validated using the probability ratio method and false discovery rate (FDR) was calculated using inverted databases and the refined method with an additional filtering for precursor mass tolerance of 12 ppm. Only peptides with a confidence of at least 95% were used to quantify the relative abundance of each peptide. Outliers at the scan and peptide levels and significant protein-abundance changes were detected from the *z*-values (the standardized variable used by the model that expresses the quantitative values in units of standard deviation) by using a FDR threshold of 5%. Results were the mean of two replicates. For transcriptomics and proteomics analysis in tick cells, the *I. scapularis* embryo-derived tick cell line ISE6, provided by Ulrike Munderloh, University of Minnesota, USA, was cultured in L-15B300 medium (Munderloh et al., 1999), except that the osmotic pressure was lowered by the addition of one-fourth sterile water by volume. The ISE6 cells were first inoculated with *A. phagocytophilum* (human NY18 isolate; Asanovich et al., 1997)-infected HL-60 cells and maintained until infection was established and routinely passaged. Uninfected and infected cultures (*N* = 3 independent cultures with ~10^7^ cells each) were sampled at 7 days post-infection (dpi; percent infected cells 71–77%; Ave ± *SD*, 74 ± 3). Transcriptomics data was obtained as described above for nymphs and adult female ticks. Two biological replicates were used for each of uninfected and infected tick cells and genes differentially expressed in response to *A. phagocytophilum* infection were selected with *P* ≤ 0.05. The proteomics analysis followed the same pipeline describe above in ticks and the MS/MS raw files generated with Xcalibur (version 2.1, Thermo Fisher Scientific) were searched against a compiled database containing all sequences from Ixodida and Anaplasmataceae (http://www.uniprot.org). Three biological replicates were used for each of uninfected and infected tick cells. For the quantitative analysis of tick proteins, after discarding *Anaplasma* proteins in infected cells, the total number of peptide-spectrum matches (PSMs) for each tick protein were normalized against the total number of PSMs in tick cells and compared between control and infected cells by Chi2-test (*P* ≤ 0.05). Although the percent of infected ticks and cultured cells was determined as described above, the bacterial load on these samples was not considered in the analysis.

The identified genes in the carbohydrate metabolic pathways were searched against the transcriptomics and proteomics data to characterize their mRNA and protein levels in response to *A. phagocytophilum* infection.

### Tertiary structure modeling and optimization

The tertiary structures of the partial protein sequences of *I. scapularis* hypoxia-inducible factor 1 alpha HIF-1α (XP_002414889) and HIF-1β (XP_002416629) proteins, which are the DNA binding N-terminus domains, were modeled using the Swiss-Model server (Biasini et al., 2014). The tertiary models were optimized using the Protein Preparation Wizard (Li et al., 2007) in the Schrödinger's Maestro software package. The Protein Preparation Wizard clusters at the highest degree of hydrogen bonding in equilibrium. Monte Carlo orientations are performed (100,000) for each cluster. The optimized structure is based on electrostatic and geometric scoring functions. Any remaining steric clashes were eliminated by minimization of the entire system with the default settings in the Schrodinger's Maestro package. The ternary structure that includes the hypoxia response element (HRE) was constructed via superimposition by using the Swiss-Model server used the mouse HIF-1α, HIF-1β, and the HRE (Dalei et al., 2015) as a template for both *I. scapularis* hypoxia-inducible protein sequences. As a final optimization step, the ternary structure (HIF-1α/HIF-1β/HRE) was processed using the normal mode ready-made script in the Metropolis Monte Carlo-based Protein Energy Landscape Exploration (PELE) server (Borrelli et al., 2005). The PELE software implements an anisotropic network model (Atilgan et al., 2001) for perturbations of the alpha-carbon backbone causing structural conformational changes. The PELE server can be accessed at https://pele.bsc.es/.

### Immunofluorescence assay in *I. scapularis* midguts and salivary glands

Female ticks fed on *A. phagocytophilum*-infected and uninfected sheep and fixed with 4% paraformaldehyde in 0.2 M sodium cacodylate buffer were embedded in paraffin and used to prepare sections on glass slides as previously described (Ayllón et al., 2015). The paraffin was removed from the sections through two washes in xylene and the sections were hydrated by successive 5 min washes with a graded series of 100, 96, and 65% ethanol and finally with distilled water. Next, the slides were treated with Proteinase K (Dako, Barcelona, Spain) for 7 min, washed with 0,1% PBS-Tween 20 (Sigma-Aldrich, St. Louis, MI, USA) and blocked with 2% bovine serum albumin (BSA; Sigma-Aldrich) in PBS-Tween 20 during 1 h at room temperature. The slides were then incubated overnight at 4°C with mouse anti-Glyceraldehyde 3-phosphate dehydrogenase (GAPDH) monoclonal antibodies (ab50567; Abcam, Cambridge, UK) diluted 1:100 in 2% BSA/PBS-Tween 20. Preimmune serum was used as control. After 3 washes with PBS-Tween 20, the slides were incubated for 1 h with rabbit anti-mouse IgG conjugated with FITC (Sigma-Aldrich) diluted 1:160 in 2% BSA/PBS-Tween 20. Finally, after two washes with PBS the slides were mounted on ProLong Diamond Antifade Mountant with DAPI reagent (Thermo Scientific, Madrid, Spain). The sections were examined using a Zeiss LSM 800 with Airyscan (Carl Zeiss, Oberkochen, Germany).

### Infected and uninfected cultured ISE6 tick cells

The *I. scapularis* embryo-derived tick cell line ISE6 were cultured as described above, infected with *A. phagocytophilum* (human NY18 isolate; Asanovich et al., 1997) or mock-infected and maintained according to Munderloh et al. (1999).

### Pharmacological studies in cultured ISE6 tick cells

*A. phagocytophilum*-infected ISE6 cells were left untreated or treated for 24 or 48 h with 5 mM 2-Deoxy-D-Glucose (ab142242, Abcam) to inhibit glycolysis (Wang et al., 2011), 100 μM LY294002 (ab120243, Abcam) to inhibit the phosphatidylinositol 3-kinase (PI3K; Sultana et al., 2010), 100 nM Chetomin (ab144222, Abcam) to inhibit the activity of HIF-1α (Misra et al., 2012) or 0.5 mM Deferoxamine mesylate (Sigma-Aldrich) to activate HIF-1α (Choi et al., 2004). After treatment, cells were harvested for the preparation of whole cell lysates to determine HIF-1α activity and for DNA extraction. Tick cell lysates were prepared by adding to cell pellets the RIPA lysis buffer (Thermo Scientific) supplemented with a protease inhibitor cocktail (cOmplete Mini, EDTA-free, Roche, Sigma-Aldrich). HIF-1α activity was determined in the cell lysate supernatants with the HIF-1 alpha Transcription Factor Assay Kit (ab133104, Abcam) following manufacturer's recommendations. *A. phagocytophilum* DNA levels were characterized by *msp4* real-time PCR normalizing against tick *16S rDNA* as described previously (Ayllón et al., 2013). Optical density values (O.D. 450 nm) for HIF-1α activity and normalized Ct values for *A. phagocytophilum* DNA levels were compared between treated and untreated control cells by Student's *t*-test with unequal variance (*P* = 0.05; *N* = 4 biological replicates).

## Results

### Major carbohydrate metabolic pathways described in other organisms are present in *I. scapularis* and are affected by *A. phagocytophilum* infection

**Figure 1:**
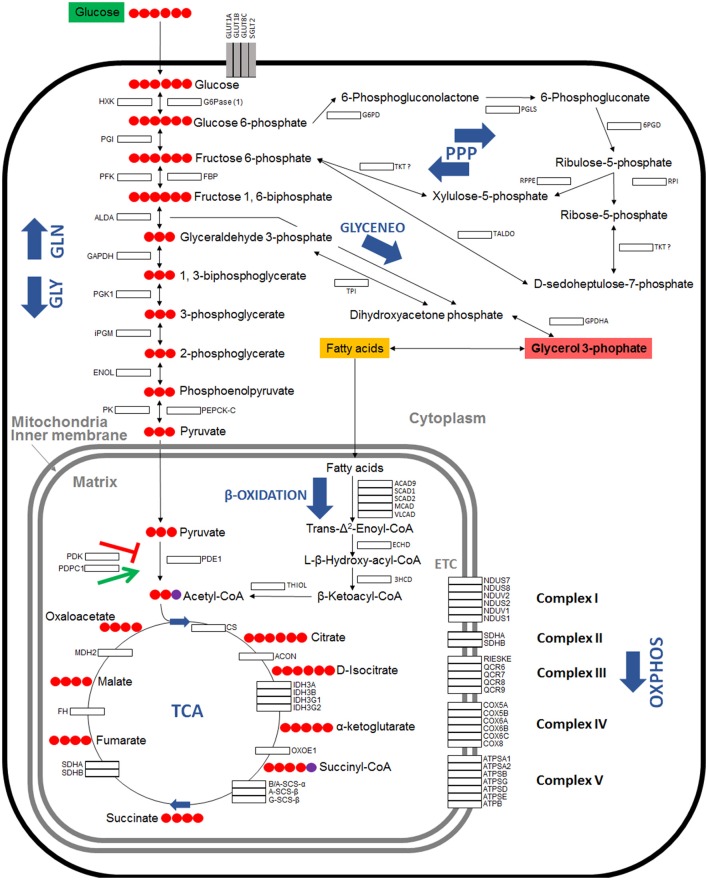
**Model of carbohydrate metabolism in ***I. scapularis*****. The main enzymes involved in Glycolysis (GLY), Gluconeogenesis (GLN), Pentose phosphate pathway (PPP), Tricarboxylic acid cycle (TCA), Oxidative phosphorylation (OXPHOS) complex I to V, β-Oxidation and Glyceroneogenesis (GLYCENEO) and present in the genome of *I. scapularis* (Table 1) are shown. The names and number of carbon molecules (Red circles) of the metabolic intermediates of these metabolic pathways are also shown. The names of the enzymes were abbreviated for the different pathways as GLY: Hexokinase (HXK), Phosphoglucose isomerase (PGI), Phosphofructokinase (PFK), Fructose-bisphosphate aldolase A (ALDA), Glyceraldehyde 3-phosphate dehydrogenase (GAPDH), Phosphoglycerate kinase 1 (PGK1), Phosphoglycerate mutase (cofactor-independent) (iPGM), Enolase (ENOL), Pyruvate kinase (PK); GLN: Glucose 6-phosphatase (1) (G6Pase (1)), Fructose-1,6-bisphosphatase (FBP), Phosphoenolpyruvate carboxykinase mitochondrial (1) (PEPCK-M (1)), Phosphoenolpyruvate carboxykinase mitochondrial (2) (PEPCK-M (2)), Phosphoenolpyruvate carboxykinase 1 cytoplasmatic (PEPCK-C), Pyruvate carboxylase (PC); PPP: Glucose 6-phosphate dehydrogenase (G6PD), 6-phosphogluconolactonase (PGLS), 6-phosphogluconate dehydrogenase (6PGD), Ribose 5-Phosphate Isomerase (RPI), Ribulose 5-Phosphate 3-Epimerase (RPPE), Transketolase (TKT), Transaldolase (TALDO); β-Oxidation: Acyl-CoA dehydrogenase family member 9 (ACAD9), Short-chain specific acyl-CoA dehydrogenase (1) (SCAD1), Short-chain specific acyl-CoA dehydrogenase (2) (SCAD2), Medium-chain acyl-CoA dehydrogenase (MCAD), Very long-chain acyl-CoA dehydrogenase (VLCAD), Enoyl-CoA hydratase (ECHD), 3-hydroxyacyl CoA dehydrogenase (3HCD), 3-ketoacyl-CoA thiolase (Thiolase I) (THIOL); GLYCENEO: Triosephosphate isomerase (TPI), Glycerol-3-phosphate dehydrogenase cytoplasmic (GPDHc); OXPHOS: Complex I: NADH dehydrogenases (NDUS7, NDUS8, NDUV2, NDUS3, NDUS2 and NDUV1), NADH-ubiquinone oxidoreductases (NDUS1, ND1 and ND5); Complex II: Succinate dehydrogenases (SDHA, SDHB, SDHC, SDHD); Complex III: Cytochrome b and b-c1 complexe subunits (QCR1, QCR2, CYTB, RIESKE, QCR6, QCR7, QCR8, QCR9, QCR10, QCR11), Cytochrome c1 (CYTC1); Complex IV: Cytochrome c oxidase subunits (COX1, COX2, COX3, COX5A, COX5B, COX6A, COX6B, COX6C, COX8); Complex V: ATP synthase subunits (ATPSA1, ATPSA2, ATPSB, ATPSG, ATPSD, ATPSE, ATPB, ATPC).

Seven major pathways involved in carbohydrate metabolism were selected for characterization (Table 1). A total of 79 genes coding for the proteins involved in glycolysis, gluconeogenesis, pentose phosphate pathway (PPP), glyceroneogenesis, TCA, mitochondrial oxidative phosphorylation (OXPHOS) and β-oxidation were identified in the *I. scapularis* genome (Table 1). Based on these results, a model for glucose metabolism in ticks was proposed (Figure 1). At least in humans, pyruvate carboxylase (PC) catalyzes the irreversible carboxylation of pyruvate to form oxaloacetate, which is then transformed in phosphoenolpyruvate by the cytoplasmic enzyme phosphoenolpyruvate carboxykinase (PEPCK-C; Berg et al., 2002). However, the PC orthologue was not identified in the *I. scapularis* genome.

**Table 1:** **Annotation of carbohydrate metabolic enzymes identified in the ***I. scapularis*** genome**.

**Enzymes**	**Abbreviation**	**Genome accession**	**GenBank ID**	**Length (amino acids)**
**GLYCOLYSIS (GLY)**				
Hexokinase	HXK	ISCW012387	EEC16047	454
Phosphoglucose isomerase	PGI	ISCW014868	EEC19698	543
Phosphofructokinase	PFK	ISCW014412	EEC18711	55
Fructose-bisphosphate aldolase A	ALDA	ISCW011371	EEC14101	364
Glyceraldehyde 3-phosphate dehydrogenase	GAPDH	ISCW018700	EEC07171	334
Phosphoglycerate kinase 1	PGK1	ISCW015616	EEC01763	415
Phosphoglycerate mutase (cofactor-independent)	iPGM	ISCW020443	EEC12432	510
Enolase	ENOL	ISCW017666	EEC06821	199
Pyruvate kinase	PK	ISCW020197	EEC12307	538
**GLUCONEOGENESIS (GLN)**				
Glucose 6-phosphatase (1)	G6Pase (1)	ISCW017459	EEC03691	356
Glucose 6-phosphatase (2)	G6Pase (2)	ISCW018612	EEC07273	282
Fructose-1,6-bisphosphatase	FBP	ISCW005292	EEC07486	338
Phosphoenolpyruvate carboxykinase mitochondrial (1)	PEPCK-M (1)	ISCW001902	EEC03297	503
Phosphoenolpyruvate carboxykinase mitochondrial (2)	PEPCK-M (2)	ISCW000524	EEC01808	288
Phosphoenolpyruvate carboxykinase 1 cytoplasmatic	PEPCK-C	ISCW001905	EEC03300	165
Phosphoenolpyruvate carboxykinase	PEPCK	ISCW008211	EEC10279	192
Pyruvate carboxylase	PC	Not found	Not found	…
**PENTOSE PHOSPHATE PATHWAY (PPP)**				
Glucose 6-phosphate dehydrogenase	G6PD	ISCW010043	EEC13253	523
6-phosphogluconolactonase	PGLS	ISCW002068	EEC04321	101
6-phosphogluconate dehydrogenase	6PGD	ISCW010101	EEC12854	507
Ribose 5-Phosphate Isomerase	RPI	ISCW012092	EEC16597	172
Ribulose 5-Phosphate 3-Epimerase	RPPE	ISCW001125	EEC01731	236
Transketolase	TKT	Not found	Not found	…
Transaldolase	TALDO	ISCW023506	EEC20008	219
**TRICARBOXYLIC ACID CYCLE (TCA CYCLE)**				
Pyruvate dehydrogenase E1	PDE1	ISCW019126	EEC09108	393
Citrate synthase	CS	ISCW009586	EEC13404	471
Aconitase	ACON	ISCW010818	EEC15463	65
Isocitrate dehydrogenase 3 α subunit	IDH3A	ISCW004116	EEC06101	362
Isocitrate dehydrogenase 3 β subunit	IDH3B	ISCW018585	EEC07698	207
Isocitrate dehydrogenase 3 γ-subunit (I)	IDH3G1	ISCW022614	EEC16834	132
Isocitrate dehydrogenase 3 γ-subunit (II)	IDH3G2	ISCW017423	EEC04192	365
2-oxoglutarate dehydrogenase E1	OXOE1	ISCW021407	EEC14367	831
GTP/ATP-specific Succinyl-CoA synthetase—α subunit	B/A-SCS-α	ISCW020686	EEC11198	217
ATP-specific Succinyl-CoA synthetase—β subunit	A-SCS-β	ISCW011750	EEC14163	425
GTP-specific Succinyl-CoA synthetase—β subunit	G-SCS-β	ISCW015999	EEC00562	422
Succinate dehydrogenase flavoprotein subunit	SDHA	ISCW000555	EEC02042	608
Succinate dehydrogenase iron-sulfur subunit	SDHB	ISCW018067	EEC06202	286
Succinate dehydrogenase cytochrome b560 subunit	SDHC	ISCW020744	EEC12476	132
Succinate dehydrogenase cytochrome b small subunit	SDHD	ISCW012012	EEC15988	208
Fumarase hydratase mitochondrial	FH	ISCW020593	EEC13182	481
Malate dehydrogenase 1 cytoplasmatic	MDH1	ISCW007624	EEC09194	302
Malate dehydrogenase 2 mitochondrial	MDH2	ISCW003528	EEC05916	340
**REGULATORY PROTEINS OF TCA CYCLE**				
Pyruvate dehydrogenase kinase	PDK	ISCW013649	EEC19175	344
Pyruvate dehyrogenase phosphatase catalytic subunit 1	PDPC1	ISCW014874	EEC19704	401
**OXIDATIVE PHOSPHORYLATION (OXPHOS) COMPLEX I (CORE SUBUNITS)**				
NADH dehydrogenase (ubiquinone) iron-sulfur protein 7	NDUS7	ISCW013042	EEC17810	162
NADH dehydrogenase (ubiquinone) iron-sulfur protein 8	NDUS8	ISCW010554	EEC13775	210
NADH dehydrogenase (ubiquinone) flavoprotein 2	NDUV2	ISCW014272	EEC18480	244
NADH dehydrogenase (ubiquinone) iron-sulfur protein 3	NDUS3	ISCW012366	EEC17298	268
NADH dehydrogenase (ubiquinone) iron-sulfur protein 2	NDUS2	ISCW017643	EEC06293	462
NADH dehydrogenase (ubiquinone) flavoprotein 1	NDUV1	ISCW005985	EEC08296	477
NADH-ubiquinone oxidoreductase 75 kDa subunit	NDUS1	ISCW003299	EEC03949	729
NADH-ubiquinone oxidoreductase chain 1	ND1	ISCW016073	EEC02134	339
NADH-ubiquinone oxidoreductase chain 2	ND2	Not found	Not found	…
NADH-ubiquinone oxidoreductase chain 3	ND3	Not found	Not found	…
NADH-ubiquinone oxidoreductase chain 4	ND4	Not found	Not found	…
NADH-ubiquinone oxidoreductase chain 4L	ND4L	Not found	Not found	…
NADH-ubiquinone oxidoreductase chain 5	ND5	ISCW016735	EEC02838	385
NADH-ubiquinone oxidoreductase chain 6	ND6	Not found	Not found	…
**OXPHOS COMPLEX II**				
Succinate dehydrogenase flavoprotein subunit	SDHA	ISCW000555	EEC02042	608
Succinate dehydrogenase iron-sulfur subunit	SDHB	ISCW018067	EEC06202	286
Succinate dehydrogenase cytochrome b560 subunit	SDHC	ISCW020744	EEC12476	132
Succinate dehydrogenase cytochrome b small subunit	SDHD	ISCW012012	EEC15988	208
**OXPHOS COMPLEX III**				
Cytochrome b-c1 complex subunit 1	QCR1	Not found	Not found	…
Cytochrome b-c1 complex subunit 2	QCR2	Not found	Not found	…
Cytochrome b	CYTB	ISCW016730	EEC02834	385
Cytochrome c1	CYTC1	ISCW016731	EEC02835	251
Cytochrome b-c1 complex subunit Rieske	RIESKE	ISCW014071	EEC19414	229
Cytochrome b-c1 complex subunit 6	QCR6	ISCW017835	EEC05133	80
Cytochrome b-c1 complex subunit 7	QCR7	ISCW012532	EEC17642	81
Cytochrome b-c1 complex subunit 8	QCR8	ISCW019560	EEC09147	82
Cytochrome b-c1 complex subunit 9	QCR9	ISCW008376	EEC10144	59
Cytochrome b-c1 complex subunit 10	QCR10	Not found	Not found	…
Cytochrome b-c1 complex subunit 11	QCR11	Not found	Not found	…
**OXPHOS COMPLEX IV**				
Cytochrome c oxidase subunit 1	COX1	Not found	ADO64507	276
Cytochrome c oxidase subunit 2	COX2	ISCW016021	EEC02091	244
Cytochrome c oxidase subunit 3	COX3	ISCW020757	EEC11360	278
Cytochrome c oxidase subunit 4	COX4	Not found	AAY66918	179
Cytochrome c oxidase subunit 5A	COX5A	ISCW008682	EEC12772	153
Cytochrome c oxidase subunit 5B	COX5B	ISCW019526	EEC10693	124
Cytochrome c oxidase subunit 6A	COX6A	ISCW019235	EEC08541	111
Cytochrome c oxidase subunit 6B	COX6B	ISCW015139	EEC20473	82
Cytochrome c oxidase subunit 6C	COX6C	ISCW016559	EEC02407	76
Cytochrome c oxidase subunit 7	COX7	Not found	Not found	…
Cytochrome c oxidase subunit 8	COX8	ISCW024928	EEC20083	68
**OXPHOS COMPLEX V (ATP SYNTHASE: F1-ATP SYNTHASE STRUCTURE/F0-ATP SYNTHASE STRUCTURE)**				
ATP synthase subunit α 1 (F1)	ATPSA1	ISCW011988	EEC16574	555
ATP synthase subunit α 2 (F1)	ATPSA2	ISCW019217	EEC08626	355
ATP synthase subunit β (F1)	ATPSB	ISCW012509	EEC17118	563
ATP synthase subunit γ (F1)	ATPSG	ISCW023477	EEC19169	314
ATP synthase subunit δ (F1)	ATPSD	ISCW018418	EEC06183	168
ATP synthase subunit ϵ (F1)	ATPSE	ISCW022265	EEC17228	55
ATP synthase subunit A (F0)	ATPA	Not found	Not found	…
ATP synthase subunit B (F0)	ATPB	ISCW012060	EEC15991	264
ATP synthase subunit C (F0)	ATPC	ISCW010495	EEC14551	152
**β-OXIDATION**				
Acyl-CoA dehydrogenase family member 9	ACAD9	ISCW010038	EEC13248	433
Short-chain specific acyl-CoA dehydrogenase (1)	SCAD1	ISCW013995	EEC19715	76
Short-chain specific acyl-CoA dehydrogenase (2)	SCAD2	ISCW002863	EEC02614	410
Medium-chain acyl-CoA dehydrogenase	MCAD	ISCW001274	EEC01138	275
Very long-chain acyl-CoA dehydrogenase	VLCAD	ISCW024772	EEC16420	254
Enoyl-CoA hydratase	ECHD	ISCW012475	EEC16250	290
3-hydroxyacyl CoA dehydrogenase	3HCD	ISCW014460	EEC18745	310
3-ketoacyl-CoA thiolase (Thiolase I)	THIOL	ISCW022661	EEC17467	406
**GLYCERONEOGENESIS**				
Triosephosphate isomerase	TPI	ISCW008869	EEC13429	247
Glycerol-3-phosphate dehydrogenase cytoplasmic	GPDHc	ISCW005254	EEC07468	340

**Figure 2:**
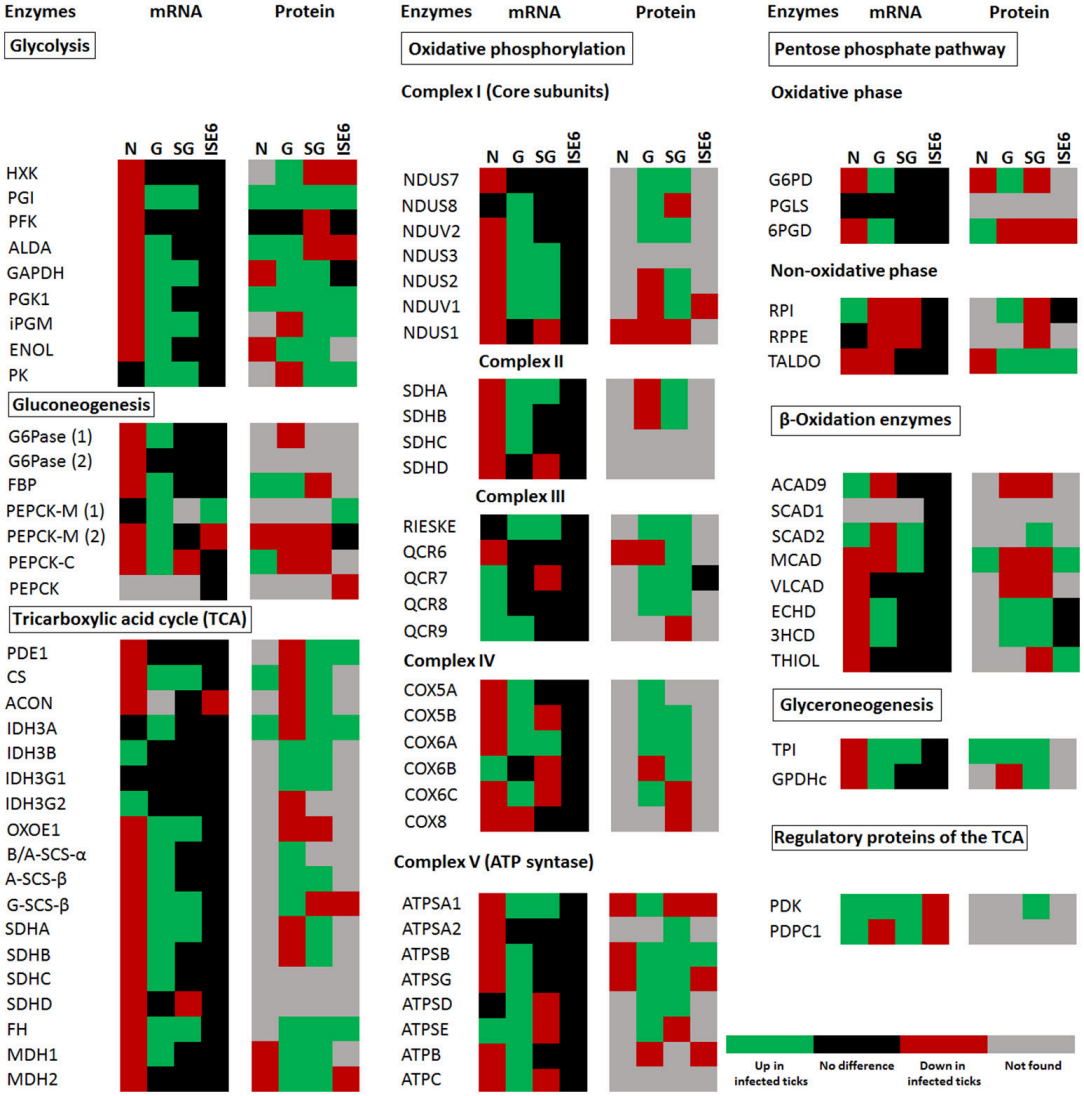
*****I. scapularis*** carbohydrate metabolism enzymes mRNA and protein levels in response to ***A. phagocytophilum*** infection**. Comparison of carbohydrate metabolism enzymes mRNA and protein levels in *I. scapularis* nymphs (N), female midguts (G), female salivary glands (SG), and ISE6 cells (ISE6) in response to *A. phagocytophilum* infection. Transcriptomics and proteomics data were obtained from previously published datasets available on the Dryad repository database, NCBI's Gene Expression Omnibus database and ProteomeXchange Consortium via the PRIDE partner repository with the dataset identifier PXD002181 and doi: 10.6019/PXD002181 (Ayllón et al., 2015; Villar et al., 2015). Name of enzymes are abbreviated as in Table 1 and Figure 1.

The carbohydrate metabolic response to *A. phagocytophilum* infection was then characterized using the quantitative transcriptomics and proteomics data generated from uninfected and *A. phagocytophilum*-infected *I. scapularis* ticks and ISE6 cultured cells (Ayllón et al., 2015; Villar et al., 2015). Most of the identified carbohydrate metabolism genes were differentially regulated in response to *A. phagocytophilum* infection in at least one of the analyzed tick tissues (Figure 2). Twenty-eight (35%), 23 (29%), 62 (78%), and 64 (81%) carbohydrate metabolism components were identified in both transcriptome and proteome of ISE6 cells, nymphs, adult female midguts, and salivary glands, respectively (Figure 2). Of these genes, 59 (75%), 14 (18%), 60 (76%), and 41 (52%) were up-regulated, while 20 (25%), 65 (82%), 16 (20%), and 35 (44%) were down-regulated in response to infection in ISE6 cells, nymphs, adult female midguts, and salivary glands, respectively (Figure 2). Many of the carbohydrate metabolism proteins were not identified by mass spectrometry (Figure 2), but the results showed similar differential regulation at the mRNA and protein levels for 50% of the identified proteins.

**Figure 3:**
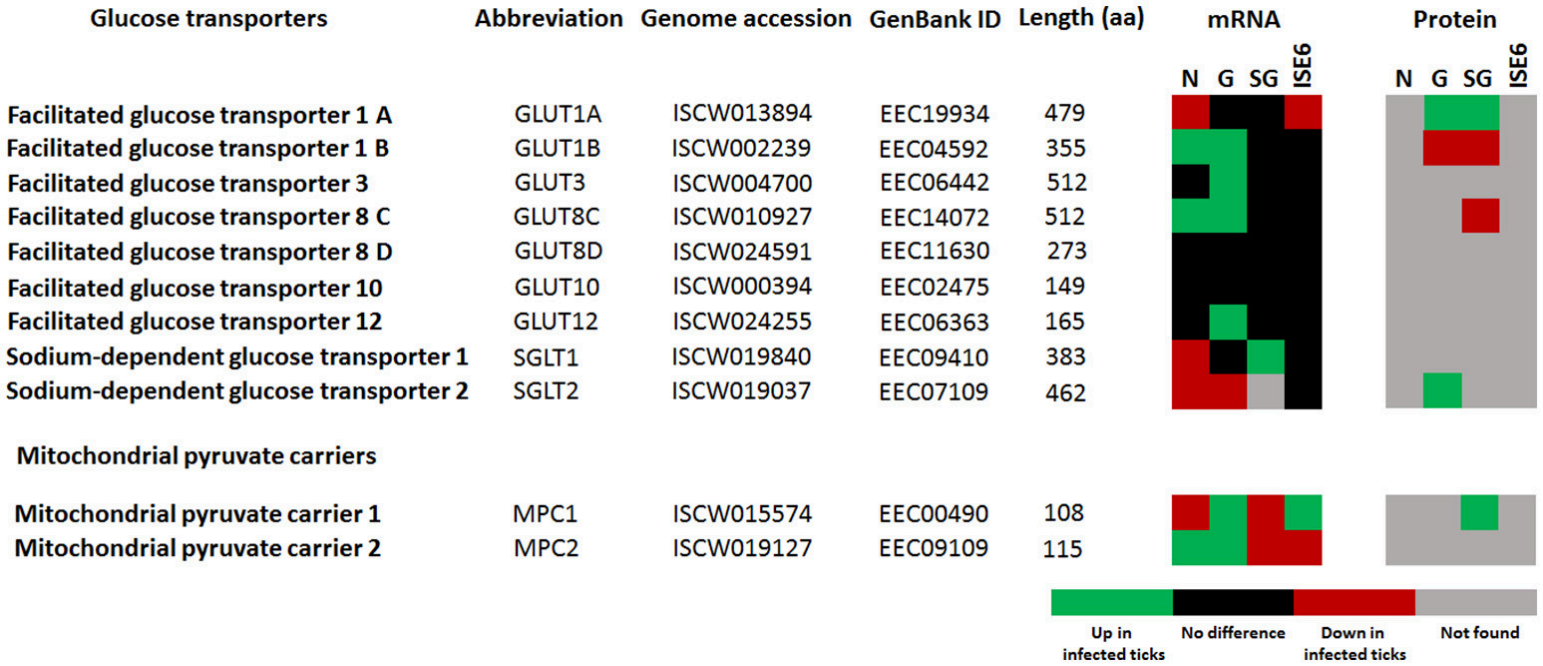
*****I. scapularis*** glucose transporters mRNA and protein levels in response to ***A. phagocytophilum*** infection**. Comparison of glucose transporters mRNA and protein levels in *I. scapularis* nymphs (N), female midguts (G), female salivary glands (SG) and ISE6 cells (ISE6) in response to *A. phagocytophilum* infection. Transcriptomics and proteomics data were obtained from previously published datasets available on the Dryad repository database, NCBI' Gene Expression Omnibus database and ProteomeXchange Consortium via the PRIDE partner repository with the dataset identifier PXD002181 and doi: 10.6019/PXD002181 (Ayllón et al., 2015; Villar et al., 2015).

Glucose transporters, and mainly facilitative glucose transporters (GLUT) are an important component of the glycolytic pathway because they transport glucose from the extracellular space to the cellular cytoplasm, which makes the glucose accessible to hexokinase (HXK; Augustin, 2010; Li et al., 2015). We found 11 putative glucose transporters in the genome of *I. scapularis*, 2 members (*sglt1* and *sglt2*) of the sodium-glucose linked transporter family (SGLT), and 9 members (2 isoforms of *glut1, glut3*, 4 isoforms of *glut8, glut10*, and *glut12*) of the GLUT family. In response to *A. phagocytophilum* infection, *glut1B, glut3, glut8C, glut10, glut12*, and *sglt1* genes were up-regulated in tick midguts, and the GLUT1A and SGLT2 proteins were over-represented in infected midguts when compared to uninfected controls (Figure 3).

As in previous experiments (Villar et al., 2015), these results supported a role for carbohydrate metabolism during *A. phagocytophilum* infection in *I. scapularis*, and suggested tissue-specific differences in response to infection.

### *A. phagocytophilum* infection activates the glycolysis pathway, but reduces gluconeogenesis and the TCA cycle in tick midguts

**Figure 4:**
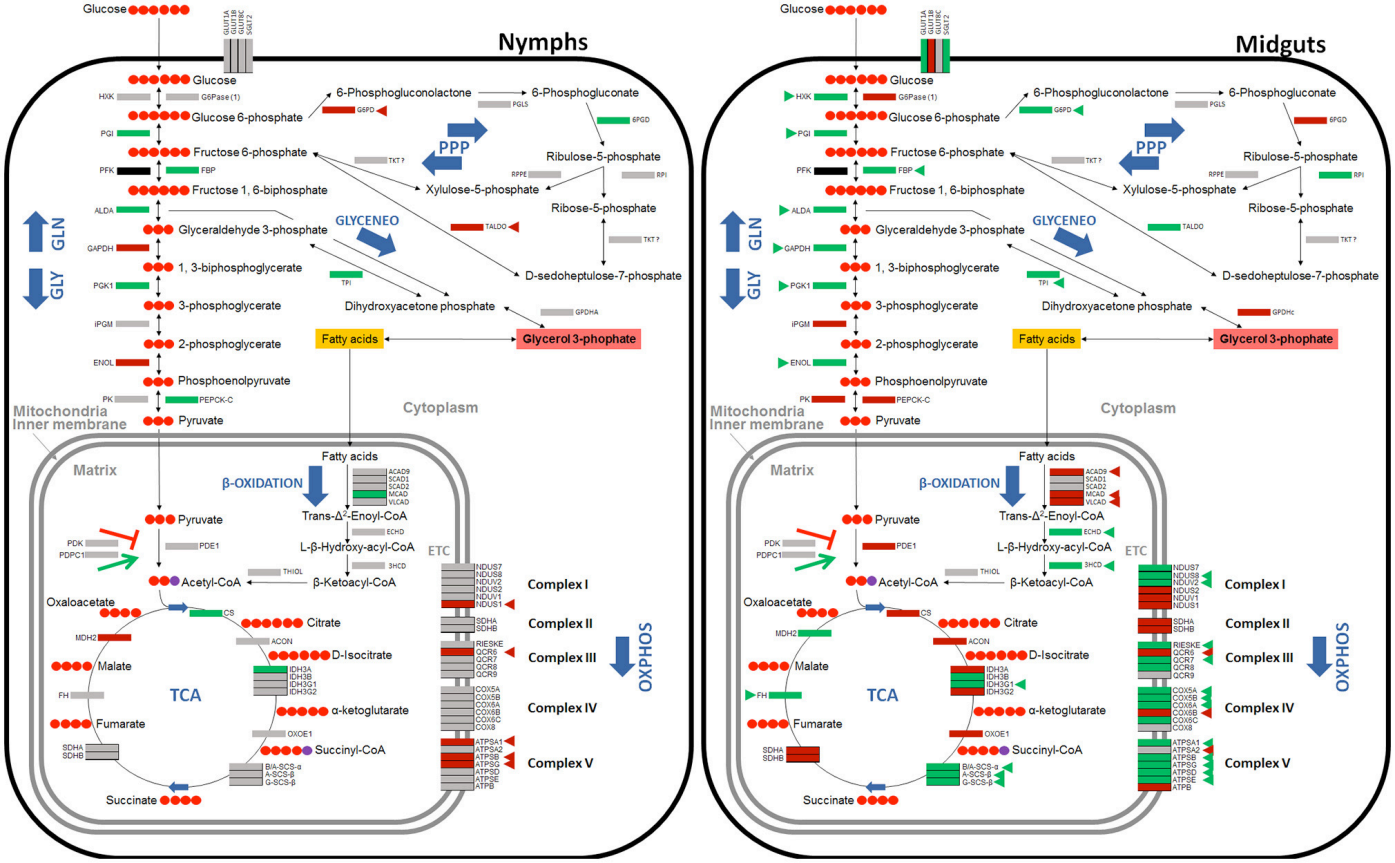
**Carbohydrate metabolic pathways in response to ***A. phagocytophilum*** infection in ***I. scapularis*** nymphs and female midguts**. Protein representation of enzymes of the *I. scapularis* carbohydrate metabolic pathways in response to *A. phagocytophilum* infection is shown. Similar changes in mRNA and protein levels are highlighted (triangles). Code: green, up-regulated/over-represented; red, down-regulated/under-represented.

**Figure 5:**
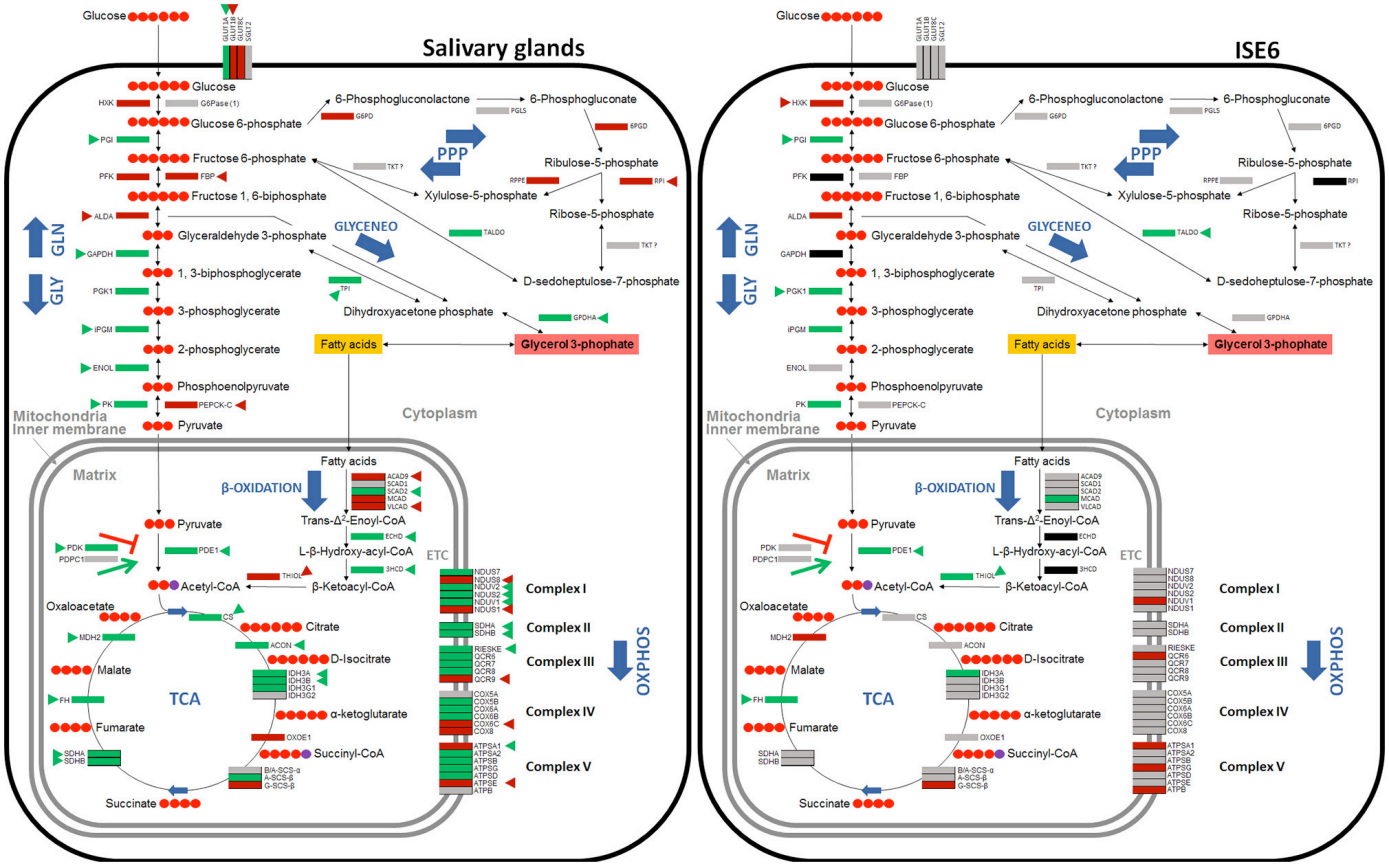
**Carbohydrate metabolic pathways in response to ***A. phagocytophilum*** infection in ***I. scapularis*** salivary glands and ISE6 cells**. Protein representation of enzymes of the *I. scapularis* carbohydrate metabolic pathways in response to *A. phagocytophilum* infection is shown. Similar changes in mRNA and protein levels are highlighted (triangles). Code: green, up-regulated/over-represented; red, down-regulated/under-represented.

Based on these results, the putative glucose metabolic pathways affected by *A. phagocytophilum* infection in different *I. scapularis* tissues and ISE6 cells were proposed (Figures 4, 5). The results showed that the genes involved in glycolysis were all up-regulated in tick midguts after *A. phagocytophilum* infection, except for phosphofructokinase (*pfk*) and *hxk* genes that did not change in response to infection (Figure 2). The up-regulation of glycolytic genes correlated with protein over-representation in tick midguts, except for the cofactor-independent phosphoglycerate mutase (*ipgm*) and pyruvate kinase (*pk*) that despite gene up-regulation in infected midguts, protein was under-represented in response to infection (Figure 2). However, since iPGM catalyzes a reversible reaction, it is not a site of major regulatory mechanisms for the glycolytic pathway. In contrast, HXK and PFK proteins, which catalyze irreversible steps of glucose glycolysis and therefore constitute major regulatory steps of this pathway, were over-represented and did not change, respectively in response to infection of tick midguts (Figure 4). The expression of the p53 target TP53-inducible glycolysis and apoptosis regulator (*tigar*, ISCW020485), which was recently shown to inhibit glycolysis (Bensaad et al., 2006), was down-regulated in tick nymphs, midguts, salivary glands, and ISE6 cells. The over-representation of all glycolytic enzymes correlated with up-regulation of their respective genes, suggesting transcriptional regulation. These data suggested that *A. phagocytophilum* infection activates glucose uptake and degradation in *I. scapularis* midguts. In agreement with an increase in glucose catabolism, the levels of glucose were significantly reduced (0.51 ± 0.06 vs. 0.31 ± 0.05, *p* < 0.005) in infected ISE6 cells when compared to uninfected controls (Villar et al., 2015).

PK catalyzes the final irreversible step of glycolysis, the transfer of a phosphate group from phosphoenolpyruvate to ADP, yielding one molecule of pyruvate and one molecule of adenosine 5′-triphosphate (ATP; Li et al., 2015). This gene was up-regulated, but protein was under-represented in response to infection in tick midguts (Figures 2, 4). Due to the regulatory role of PK, low levels of this enzyme may hamper the flux of pyruvate toward the mitochondrial matrix, decreasing the activity of TCA cycle. PK generates pyruvate, which is transported to the mitochondrial matrix by mitochondrial pyruvate carriers 1 and 2 (MPC1 and MPC2). The homologs for *mpc1* and *mpc2* genes were identified in *I. scapularis* and were up-regulated in tick midguts, but down-regulated in salivary glands in response to *A. phagocytophilum* infection (Figure 3). In the mitochondrial matrix, pyruvate is decarboxylated by pyruvate dehydrogenase E1 (PDE1; Berg et al., 2002). The decarboxylation of pyruvate is the first step of a series of enzymatic reactions that transform pyruvate into acetyl coenzyme A (acetyl-Coa), which is oxidized to CO_2_ and water in the TCA cycle (Berg et al., 2002). PDE1 is an important regulator of the TCA cycle (Berg et al., 2002). Pyruvate dehydrogenase kinase 1 (PDK1) inhibits, while pyruvate dehydrogenase phosphatase catalytic subunit 1 (PDPC1) activates PDE1 (Berg et al., 2002). We found that in the midguts of infected ticks, PDE1 was under-represented and the genes *pdk1* and *pdpc1* were up-regulated and down-regulated, respectively in response to infection (Figure 2). This result strongly suggested that *A. phagocytophilum* infection inhibited the TCA cycle in *I. scapularis* midguts. In agreement with this suggestion, several TCA cycle enzymes such as citrate synthase (CS), aconitase (ACON), isocitrate dehydrogenase [subunits α (IDH3A) and γ (IDH3G1)], 2-oxoglutarate dehydrogenase E1 (OXOE1), succinate dehydrogenase (flavoprotein subunit (SDHA), and iron-sulfur subunit (SDHB) were under-represented in midguts from infected ticks when compared to uninfected controls (Figures 2, 4).

The NADH generated by the TCA cycle is fed into the OXPHOS pathway to produce ATP (Li et al., 2015). The mRNA and protein levels of components of the OXPHOS complex I–IV present in *I. scapularis* were examined. Higher mRNA and protein levels were found for most of the respiratory components in infected tick midguts when compared to uninfected controls (Figure 2). This result suggested that ticks might have a regulatory mechanism to maintain the levels of ATP in response to the inhibition of the TCA cycle.

Finally, we found that although most genes were up-regulated, all proteins of gluconeogenesis (synthesis of glucose from non-carbohydrate precursors) were under-represented in tick midguts, except for fructose-1,6-bisphosphatase (FBP; Figure 2). FBP catalyzes an irreversible reaction in the gluconeogenesis, the conversion of fructose 1,6-bisphosphate into fructose 6-phosphate, and therefore it is a regulatory step of this anabolic pathway. However, the enzyme glucose 6-phosphatase that catalyzes the last step of gluconeogenesis, the transformation of glucose 6-phosphate into free glucose was under-represented. These results suggested that midgut cells in *A. phagocytophilum*-infected ticks tend to keep glucose 6-phosphate within the cells for other metabolic processes (e.g., glycogen synthesis).

### HIF-1 components are up-regulated in *I. scapularis* infected with *A. phagocytophilum*

HIF-1 is a heterodimeric transcription factor consisting of a constitutively expressed β-subunit (HIF-1β) and an oxygen-regulated α-subunit (HIF-1α; Pagé et al., 2002; Déry et al., 2005; Ziello et al., 2007; Badeaux and Shi, 2013). The gene encoding for HIF-1α (ISCW023657), which is a major transcriptional activator of glycolytic genes (Hu et al., 2006), was significantly up-regulated in tick midguts, but not in nymphs, salivary glands or ISE6 cells in response to *A. phagocytophilum* infection (Ayllón et al., 2015; Villar et al., 2015). The expression of the gene encoding for the HIF-1β (ISCW023999), which is a molecular partner of HIF-1α, was up-regulated in midguts from infected ticks, but was down-regulated in nymphs and did not change in salivary glands and ISE6 cells in response to infection (Ayllón et al., 2015; Villar et al., 2015).

**Figure 6:**
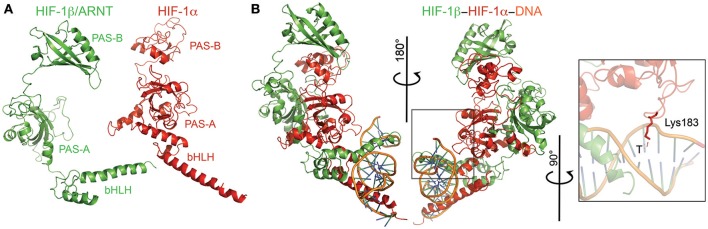
**Modeling of the HIF-1α-HIF-1β-HRE complex**. **(A)** HIF-1α (red) and HIF-1β (green) monomers with their respective domains labeled. **(B)** Ternary complex of both monomers plus the hypoxia response element (HRE; orange). The inset shows the conserved Lys residue that interacts with the thymine (T) of HRE.

Both *I. scapularis* HIF-1α and HIF-1β (a.k.a, aryl hydrocarbon receptor nuclear translocator or ARNT) possess the conserved basic helix-loop-helix (bHLH) domain and two PAS domains (Figures 6A,B). The bHLH domain of HIF-1α and HIF-1β were highly conserved with identical interacting residues as those previously identified (Dalei et al., 2015). The PAS-B domain of HIF-1α was disordered due to its low conservation and the presence of small insertions and deletions when compared to the mouse HIF-1α (Dalei et al., 2015). The PAS-B of HIF-1α interacts with both the PAS domain of HIF-1β, thereby making it difficult to determine interacting residues. One key feature of HIF-1α is the presence of two residues lysine (Lys) and glycine (Gly), which interact with the nucleotides of hypoxia response element (HRE; Dalei et al., 2015). The *I. scapularis* HIF-1α had the conserved Lys, but showed an asparagine (Asn) to Gly substitution (Figure 6).

### The PI3K/Akt pathway is present in *I. scapularis* and regulated in response to *A. phagocytophilum* infection

It has been demonstrated that a number of non-hypoxic stimuli are highly capable of inducing the expression of *hif-1* (Pagé et al., 2002; Déry et al., 2005). The PI3K pathway and its downstream effectors, mTOR and p70S6 kinase (p70S6K), may induce a hypoxia-independent increase in HIF-1α levels (Déry et al., 2005). In addition, diacylglycerol (DAG)-sensitive protein kinases C (PKC) were shown to up-regulate *hif-1*α gene expression in a hypoxia-independent manner (Pagé et al., 2002; Déry et al., 2005). Infection with *A. phagocytophilum* activates PI3K in *I. scapularis* (Sultana et al., 2010). Therefore, we characterized the mRNA and protein levels of tick PI3K components in response to *A. phagocytophilum* infection.

**Figure 7:**
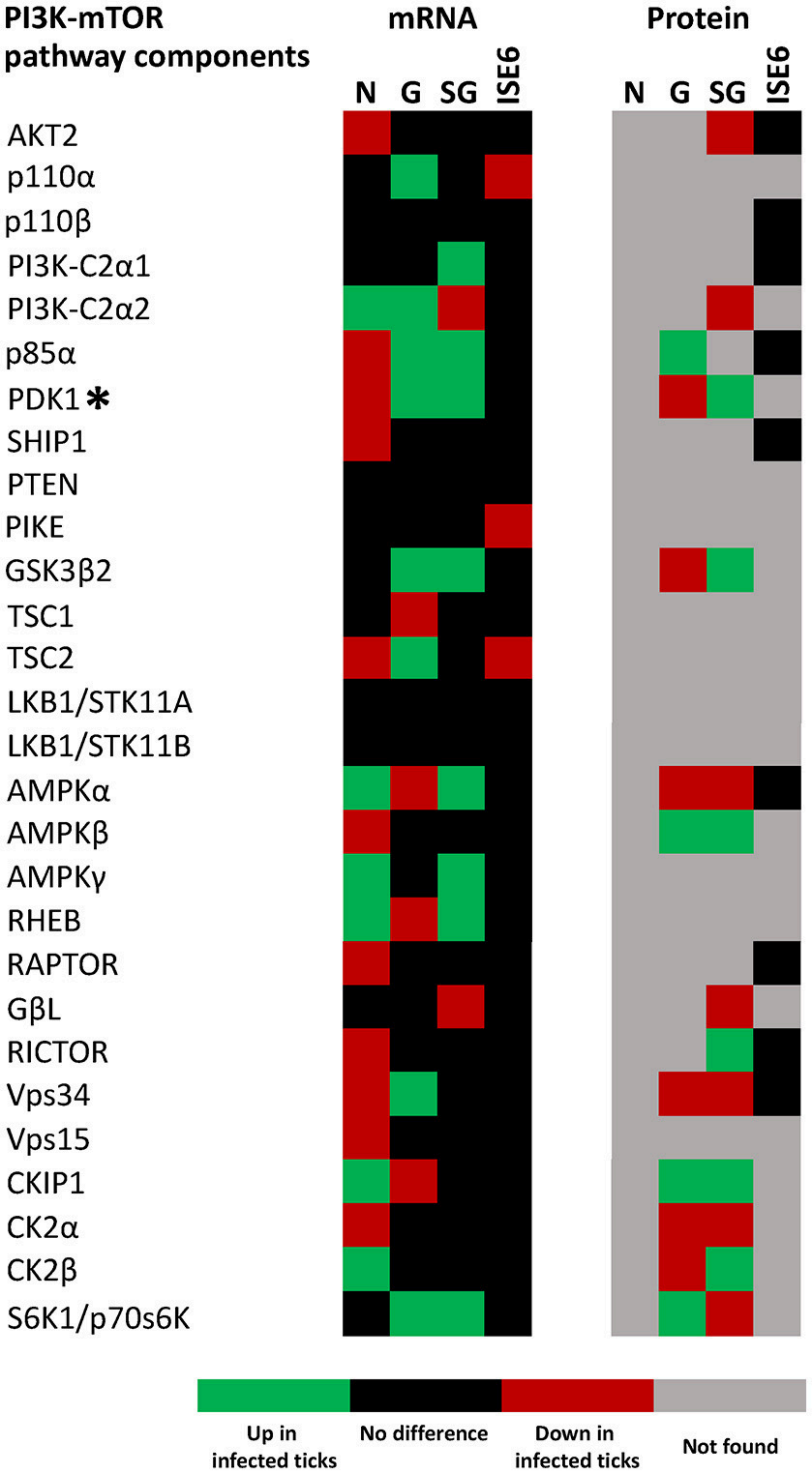
*****I. scapularis*** PI3K-mTOR pathway components mRNA and protein levels in response to ***A. phagocytophilum*** infection**. Comparison of PI3K-mTOR pathway components mRNA and protein levels in *I. scapularis* nymphs (N), female midguts (G), female salivary glands (SG) and ISE6 cells (ISE6) in response to *A. phagocytophilum* infection. Transcriptomics and proteomics data were obtained from previously published datasets available on the Dryad repository database, NCBI's Gene Expression Omnibus database and ProteomeXchange Consortium via the PRIDE partner repository with the dataset identifier PXD002181 and doi: 10.6019/PXD002181 (Ayllón et al., 2015; Villar et al., 2015). Name of enzymes are abbreviated as in Table 2. (^*^) In this figure, PDK1 stands for 3- phosphoinositide-dependent protein kinase 1, and not Pyruvate dehydrogenase kinase 1, which is also abbreviated as PDK1.

Most PI3K-mTOR pathway components found in other organisms (Pilot-Storck et al., 2010) were identified in the *I. scapularis* genome (Table 2). The tick PI3K components were differentially regulated in response to *A. phagocytophilum* infection (Figure 7). The *p70S6K* and PI3K components (*p85*α and *p110*α) were up-regulated in tick midguts and salivary glands in response to infection. Additionally, p70S6K and p85α proteins were over-represented in midguts from infected ticks when compared to uninfected controls (Figure 7). Oxygen-independent activation of *hif-1*α may involve PI3K components, p70S6K and mTOR (Déry et al., 2005). However, we did not find an mTOR ortholog in the *I. scapularis* genome. DAG-sensitive PKCs were also suggested to play a role in oxygen-independent activation of *hif-1*α (Pagé et al., 2002). Protein PKCα was found to be over-represented in tick midguts and salivary glands, while the *PKC*ε gene was up-regulated in tick midguts in response to infection.

**Table 2:** **Annotation of PI3K-mTOR pathway components identified in the ***I. scapularis*** genome**.

**Proteins**	**Abbreviation**	**Genome accession**	**GenBank ID**	**Length (amino acids)**
RAC-alpha serine/threonine-protein kinase	AKT2	ISCW018284	EEC06033	371
Phosphatidylinositol 4,5-bisphosphate 3-kinase catalytic subunit α	p110α	ISCW014229	EEC18977	617
Phosphatidylinositol 4,5-bisphosphate 3-kinase catalytic subunit β	p110β	ISCW010469	EEC14480	491
Phosphatidylinositol-4-phosphate 3-kinase C2 domain-containing α (1)	PI3K-C2α1	ISCW022811	EEC17358	1638
Phosphatidylinositol-4-phosphate 3-kinase C2 domain-containing α (2)	PI3K-C2α2	ISCW004026	EEC06376	1142
Phosphatidylinositol 3-kinase regulatory subunit α	p85α	ISCW011707	EEC13970	427
3- phosphoinositide-dependent protein kinase 1	PDK1	ISCW021115	EEC15400	521
Phosphatidylinositol 3,4,5-trisphosphate 5-phosphatase 1	SHIP1	ISCW000015	EEC00572	535
Phosphatidylinositol 3,4,5-trisphosphate 3-phosphatase PTEN	PTEN	ISCW021211	EEC15363	363
Arf-GAP with GTPase, ANK repeat and PH domain-containing protein	PIKE	ISCW014072	EEC18950	558
Glycogen synthase kinase 3 β (2)	GSK3β2	ISCW009451	EEC11886	588
Hamartin	TSC1	ISCW023319	EEC19233	874
Tuberin	TSC2	ISCW012320	EEC16560	429
Serine/threonine kinase 11 (1)	LKB1/STK11A	ISCW024898	EEC19357	170
Serine/threonine kinase 11 (2)	LKB1/STK11B	ISCW015294	EEC20092	293
AMP-activated protein kinase α	AMPKα	ISCW020637	EEC13103	510
AMP-activated protein kinase β	AMPKβ	ISCW021036	EEC15614	183
AMP-activated protein kinase γ	AMPKγ	ISCW010164	EEC13342	154
Ras homolog enriched in brain	RHEB	ISCW018020	EEC05182	182
Regulatory-associated protein of mTOR	RAPTOR	ISCW018753	EEC07385	995
G protein β subunit-like	GβL	ISCW002765	EEC03813	324
Rapamycin-insensitive companion of mTOR	RICTOR	ISCW018420	EEC06185	987
Phosphatidylinositol 3-kinase catalytic subunit type 3	Vps34	ISCW008679	EEC12769	864
Serine/threonine-protein kinase VPS15	Vps15	ISCW017661	EEC06819	1351
Pleckstrin homology domain-containing, family O member 1	CKIP1	ISCW021605	EEC15223	359
Casein kinase 2 subunit α	CK2α	ISCW007259	EEC10594	436
Casein kinase 2 subunit β	CK2β	ISCW014459	EEC18744	222
Ribosomal protein S6 kinase beta-1 (p70s6 kinase)	S6K1/p70s6K	ISCW017390	EEC03348	344

### Functional studies support a role for carbohydrate metabolism during *A. phagocytophilum* infection of tick cells

**Figure 8:**
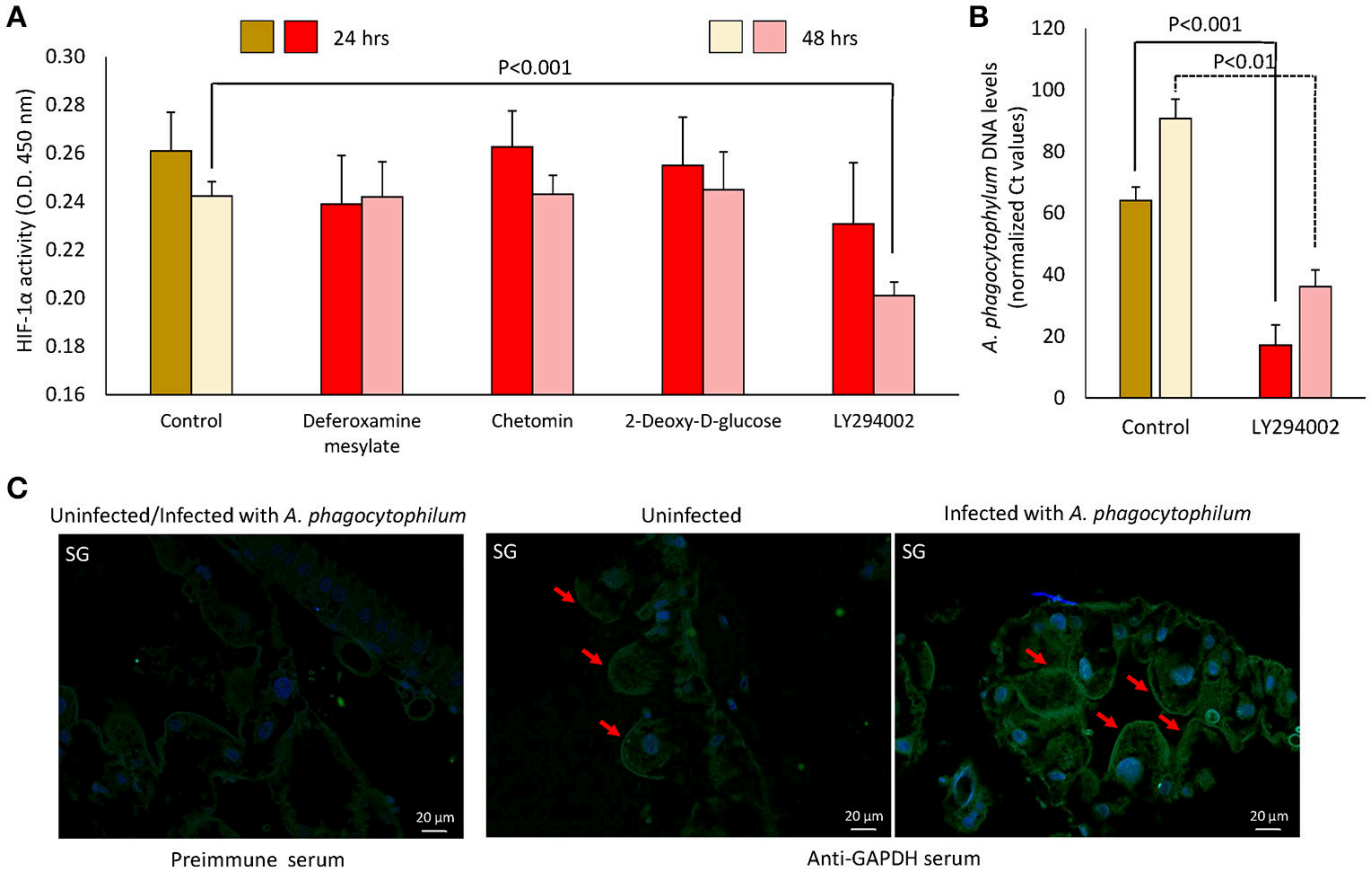
**Functional analysis supports a role for glycolysis during ***A. phagocytophilum*** infection of tick cells. (A)** *A. phagocytophilum*-infected ISE6 cells were left untreated (Control) or treated for 24 or 48 h with 2-Deoxy-D-Glucose to inhibit glycolysis, LY294002 to inhibit the PI3K, Chetomin to inhibit the activity of HIF-1α or Deferoxamine mesylate to activate HIF-1α. HIF-1α activity was determined in cell lysates, and O.D. 450 nm values were shown as average + *SD* and compared between treated and untreated control cells by Student's *t*-test with unequal variance (*P* = 0.05; *N* = 4 biological replicates). **(B)** *A. phagocytophilum* DNA levels were characterized in LY294002-treated and untreated control cells by *msp4* real-time PCR normalizing against tick *16S rDNA*. Normalized Ct values were compared between treated and untreated control cells by Student's *t*-test with unequal variance (*P* < 0.01; *N* = 4 biological replicates). **(C)** Representative images of imunofluorescence analysis of uninfected and *A. phagocytophilum*-infected adult female *I. scapularis* salivary gland acini (SG). Tick tissues were stained with preimmune control serum or mouse anti-GAPDH monoclonal antibodies (green, FITC) or DAPI (blue) and superimposed (FITC+DAPI). Bars, 20 μm. Red arrows illustrate the positive staining for GAPDH in tick SG.

Functional studies were focused on glycolysis by targeting the process at different levels (Figures 8A,B). *A. phagocytophilum*-infected ISE6 cells were left untreated or treated for 24 or 48 h with 2-Deoxy-D-Glucose to inhibit glycolysis (Wang et al., 2011), LY294002 to inhibit the PI3K (Sultana et al., 2010), Chetomin to inhibit the activity of HIF-1α (Misra et al., 2012) or Deferoxamine mesylate to activate HIF-1α (Figures 8A,B). Both HIF-1α (Ayllón et al., 2015; Villar et al., 2015), which is a major transcriptional activator of glycolytic genes (Hu et al., 2006), and most PI3K components (Figure 7) were affected in response to *A. phagocytophilum* infection. The results showed that LY294002 was the only compound active in ISE6 tick cells (Figure 8A). The lack of effect of chetomin and Deferoxamine mesylate on tick HIF-1α activity may be due to structural differences between tick and mammalian HIF-1α. The treatment of tick cells with LY294002 for 48 h resulted in lower HIF-1α activity when compared to untreated control cells (Figure 8A). Treatment with LY294002 has been shown in *I. scapularis* and other organisms to inhibit the PI3K pathway (Sultana et al., 2010), which is involved in the induction of HIF-1α to activate the glycolysis (Déry et al., 2005). Therefore, the results obtained in tick cells supported a role for PI3K in HIF-1α induction in tick cells. The decrease in *A. phagocytophilum* infection after LY294002 treatment for 24 and 48 h (Figure 8B) provided additional support to these results. As expected from the lack of effect on HIF-1α activity after incubation of tick cells with the other compounds, treatment did not affect *A. phagocytophilum* infection in these cells. Additionally, the immunofluorescence assay of GAPDH, which was over-represented in *I. scapularis* midguts and salivary glands in response to *A. phagocytophilum* infection (Figures 4, 5), corroborated the results of the proteomics analysis in tick salivary glands (Figure 8C).

## Discussion

Recently, Villar et al. (2015) showed modifications in tick cell glucose metabolism during *A. phagocytophilum* infection. The results evidenced that infection affected the glucose metabolic pathway in tick cells through phosphoenolpyruvate carboxykinase (PEPCK) inhibition leading to decreased gluconeogenesis, which also results in the inhibition of cell apoptosis that increases pathogen infection of tick cells (Villar et al., 2015). Furthermore, these results provided evidence that other carbohydrate metabolic pathways are also affected by pathogen infection. Therefore, the objective of this study was the characterization of the dynamics of major carbohydrate pathways during *A. phagocytophilum* infection of *I. scapularis*.

Cellular glycolysis converts glucose to pyruvate, which enters the mitochondria where it is converted into acetyl-CoA, and is metabolized via the TCA cycle yielding reducing equivalents that are used for OXPHOS to generate ATP (Eisenreich et al., 2013, 2015). TCA cycle can be also fueled by the acetyl-CoA produced via degradation of fatty acids after β-Oxidation (Eisenreich et al., 2013). Alternatively, glucose can be used to produce Ribose 5-phosphate, a precursor for the synthesis of nucleotides through the PPP (Berg et al., 2002; Figure 1). In addition, glyceroneogenesis uses glycerol 3-phophate, obtained from the reduction of dihydroxyacetone phosphate (glycolytic intermediate) to synthetize fatty acids, and therefore is a major link between carbohydrate metabolism and lipid metabolism (Berg et al., 2002; Figure 1).

In this study, orthologs for 79 components of major carbohydrate metabolic pathways were identified in *I. scapularis*, and their role was characterized in response to *A. phagocytophilum* infection. The analysis was focused on seven pathways including glycolysis, gluconeogenesis, PPP, glyceroneogenesis, β-Oxidation, TCA cycle, and OXPHOS. The results showed that genes involved in glycolysis were up-regulated in *I. scapularis* ticks infected with *A. phagocytophilum*. In contrast, PEPCK, which is the enzyme that catalyzes the first step of gluconeogenesis (after this step the molecules are “committed” to the pathway and will ultimately end up in the pathway's final product) was under-represented (Figure 2). However, in addition to low levels of PEPCK, low TCA cycle activity might be also necessary to control gluconeogenesis (Burgess et al., 2007). Interestingly, we found that several enzymes and intermediates such as succinate of the TCA cycle are also under-represented in infected tick ISE6 cells and midguts (Figures 4, 5; Villar et al., 2015). These results strongly suggested that upon *A. phagocytophilum* infection, glycolysis is enhanced, TCA cycle inhibited and in agreement with our previous results (Villar et al., 2015), gluconeogenesis is inhibited. These findings supported the idea that *A. phagocytophilum* infection might be an energy-demanding process for the tick cells and that the pathogen may benefit from glycolytic intermediates.

Intracellular bacteria trigger diverse host metabolic responses (Eisenreich et al., 2013, 2015). Numerous transcriptome studies have identified some of these metabolic responses as unspecific (triggered by extracellular/intracellular and pathogenic/non-pathogenic bacteria) and are therefore called “core host responses” (Boldrick et al., 2002). These “core host responses” are modulated in different ways by virulence mechanisms of different bacteria species (Boldrick et al., 2002). For example, *in vivo* studies in mice showed that the facultative intracellular pathogen *Mycobacterium tuberculosis* induces a reduction in the levels of glucose and the TCA cycle intermediates oxaloacetate and fumarate, but also an increase in lactate and succinate concentration (Shin et al., 2011; Somashekar et al., 2011). In *Listeria monocytogenes*, Lecuit et al. (2007) found that the transcription of most glycolytic genes was enhanced after infection in mice, particularly *hxk II*. The upregulation of glycolytic genes was linked to enhanced *hif-1*α expression and downregulation of the gluconeogenic gene *fbp* (Lecuit et al., 2007). The above examples illustrate commonalities among the host metabolic responses to *M. tuberculosis, L. monocytogenes* and *A. phagocytophilum* infection.

**Figure 9:**
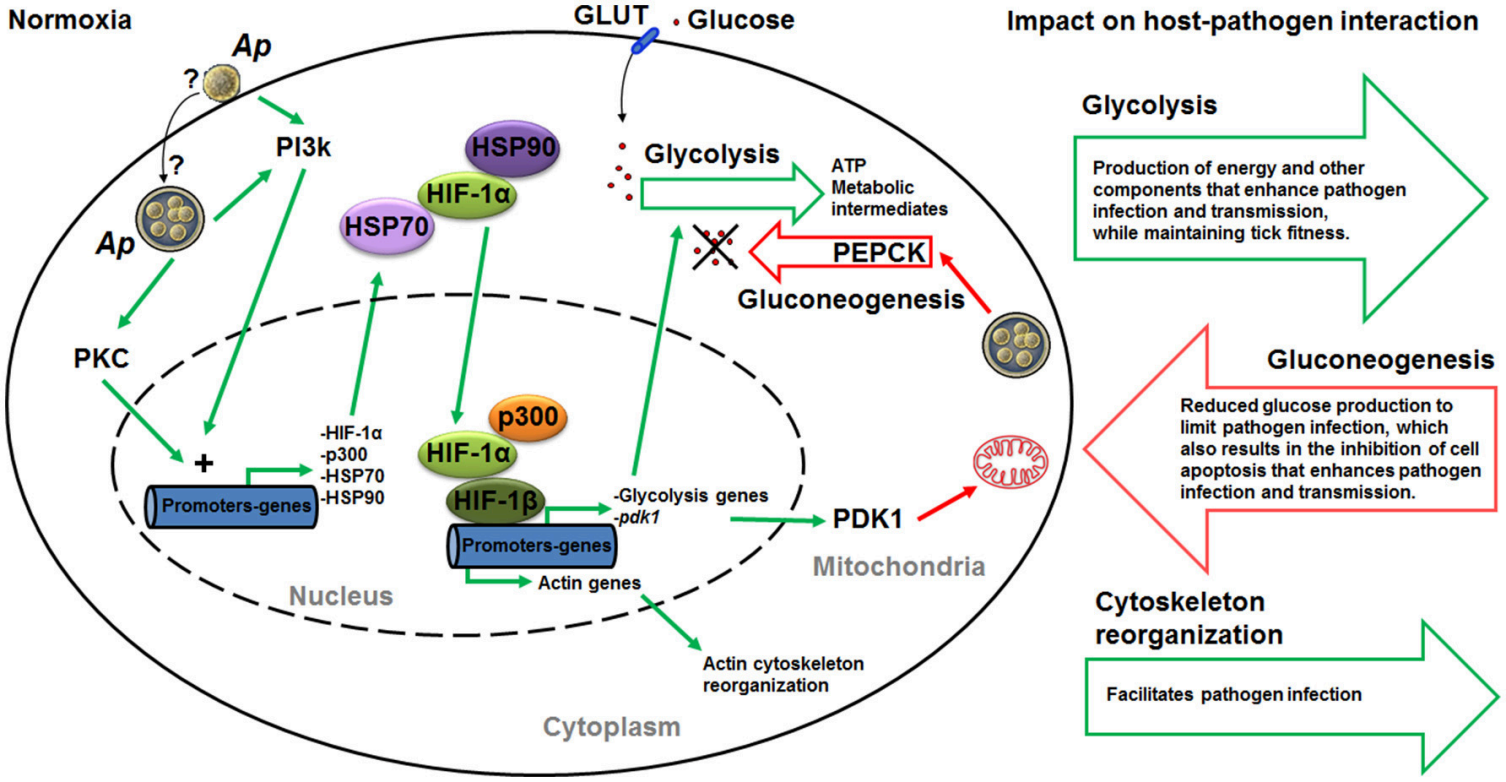
**Mechanistic model of carbohydrate metabolism manipulation by ***A. phagocytophilum*****. We propose a mechanism by which *A. phagocytophilum* enhances glycolysis through HIF activation under normoxic conditions. Upon contact with the host membrane or once inside the parasitophorous vacuole, *A. phagocytophilum* induces the activation of PI3K and PKC that ultimately induce the expression of heat shock proteins HSP70 and HSP90, the acetyltransferase p300 and *hif-1*α. In normoxia, HSP70 and HSP90 stabilize HIF-1α, making possible the recruitment of p300 and HIF-1α to form a complex with HIF-1β in the nucleus. This complex activates the expression of HIF-1α target genes, which include glycolytic genes and *pdk1*. This mechanism will result in the increase in glycolysis and inhibition of gluconeogenesis. Furthermore, HIF activation can also leads to actin cytoskeleton reorganization. Therefore, by activating the HIF system *A. phagocytophilum* may regulate at the same time the tick cell carbohydrate metabolism and cytoskeleton organization. Green and red lines indicate induction/activation and inhibition, respectively. Question marks indicate that we do not know whether *A. phagocytophilum* induces the activation of PI3K and PKC upon contact with the host membrane or once inside the parasitophorous vacuole. Ap, *A. phagocytophilum*; PI3K, phosphatidylinositol 3-kinase pathway; diacylglycerol (DAG)-sensitive protein kinases C (PKC); HIF, hypoxia-inducible factor; PDK1, pyruvate dehydrogenase kinase 1; GLUT, facilitative glucose transporters; PEPCK, phosphoenolpyruvate carboxykinase.

Based on the results of this study, a model was proposed in which *A. phagocytophilum* induces transcriptional activation of *hif-1*α through PI3K (p85α, p110α, and p70S6K) and PKC to activate the glycolytic pathway and inhibit the TCA cycle in infected ticks (Figure 9). This model was partially supported by functional studies using a PI3K inhibitor to monitor the effect on HIF-1α activity and pathogen infection (Figures 8A,B). In normoxia, HIF-1α is rapidly degraded. Therefore, *A. phagocytophilum* may have additional mechanisms to stabilize HIF-1α at normal oxygen concentrations. The increase in the levels of heat shock proteins HSP70 and HSP90 may contribute to the stabilization of HIF-1α in normoxia (Zhou et al., 2004). Previously, we showed that HSP70 and HSP90 were over-represented in *A. phagocytophilum*-infected ISE6 tick cells when compared to uninfected cells (Villar et al., 2015). Furthermore, mobilization of p300 at HIF target genes may be an additional requirement of HIF-mediated transcriptional activation (Badeaux and Shi, 2013). In agreement with this model, recently we showed that *A. phagocytophilum* induces the production of p300 to inhibit cell apoptosis and increase bacterial multiplication in tick cells (Cabezas-Cruz et al., 2016). By inhibiting the TCA cycle in host cells, *A. phagocytophilum* may inhibit the entrance of glutamine and glutamate to the TCA cycle via α-ketoglutarate, which is one of the intermediates in the TCA cycle. This process may increase the cytoplasmic concentration of glutamine and glutamate that can then be used by *A. phagocytophilum*. The TCA cycle of *A. phagocytophilum* is incomplete (because one gene for isocitrate dehydrogenase is missing from the genome) and requires the exogenous acquisition of glutamine and glutamate (Huang et al., 2007), which the pathogen may obtain from the host cell cytoplasm. *A. phagocytophilum* has the enzyme that converts glutamine to glutamate and glutamate can be then transformed to α-ketoglutarate that may fuel the bacterial TCA cycle.

Interestingly, it was recently shown that inhibition of prolyl hydroxylases, which are involved in the regulation of HIF-1α activity, induces HIF-1α-dependent cytoskeletal remodeling in endothelial cells (Weidemann et al., 2013). Therefore, it is possible that *A. phagocytophilum* activates HIF-1α through PI3K to regulate simultaneously the carbohydrate metabolism and cytoskeleton organization to facilitate infection and multiplication in tick cells (Figure 9).

The comparison of metabolic pathways between selected Anaplasmataceae showed that *Rickettsia prowazekii, Ehrlichia chaffeensis, Neorickettsia sennetsu, Wolbachia pipientis*, and *A. phagocytophilum* might not be able to actively carry out glycolysis. Therefore, glycolytic metabolic intermediates produced by host cells may be necessary for the development of these bacteria. Only those glycolysis enzymes necessary to produce glyceraldehyde-3-phosphate and dihydroxyacetone phosphate from phosphoenolpyruvate are present in the genome of these *Rickettsia* spp. (Dunning Hotopp et al., 2006). Therefore, phosphoenolpyruvate may be one of the glycolytic intermediates that *A. phagocytophilum* hijacks from the host cell cytoplasm. Additionally, glycerol-3-phosphate, which is a product of glyceroneogenesis, was proposed as another metabolite taken from the host by *Rickettsia* spp. (Dunning Hotopp et al., 2006). In agreement with this proposed model, the glyceroneogenesis enzymes triosephosphate isomerase (TPI) and glycerol-3-phosphate dehydrogenase cytoplasmic (GPDHc) were over-represented in infected tick nymphs, midguts and/or salivary glands when compared to uninfected controls.

The failure to identify *I. scapularis* orthologs for some genes may be due to the absence of these pathway components in ticks or the fact that only ~57% of the genome have been sequenced and assembled for this species (de la Fuente et al., 2016b; Gulia-Nuss et al., 2016). These results were similar to those obtained before for other genes and proteins in response to *A. phagocytophilum* infection, showing tissue-specific differences in the response to pathogen infection (Ayllón et al., 2015). As previously discussed, these results suggested that differences between mRNA and protein levels could be due to delay between mRNA and protein accumulation which requires sampling at different time points and/or the role for post-transcriptional and post-translational modifications in the tick tissue-specific response to *A. phagocytophilum* infection (Ayllón et al., 2015; Villar et al., 2015; Cabezas-Cruz et al., 2016).

## Conclusions

These results support that major carbohydrate metabolic pathways are conserved in ticks. *A. phagocytophilum* infection has a major impact on the regulation of carbohydrate metabolic pathways in tick cells. As a result of the studies reported here, a mechanism was proposed by which this pathogen might induce the expression and stabilization of HIF-1α to increase glycolysis, suppress TCA cycle to reduce gluconeogenesis, and regulate cytoskeleton organization (Figure 9). This may be achieved by a coordinated action of PI3K/PKC pathway, for induction of *hif-1*α expression, and HSP70/90 and p300, for HIF-1α stabilization. The increase in glycolysis results in the production of energy and other components that enhance pathogen infection and transmission while preserving tick fitness. The reduction in gluconeogenesis may be a cell response to limit pathogen infection, but also results in the inhibition of cell apoptosis to enhance pathogen infection and transmission. These mechanisms provided additional support for the co-evolution of tick-pathogen interactions that can produce both conflict and cooperation between them (de la Fuente et al., 2016c). The identification of these mechanisms provided additional evidences to support that *A. phagocytophilum* uses similar strategies to infect vertebrate hosts and ticks (de la Fuente et al., 2016a), therefore suggesting the possibility of developing strategies for a more effective control of *A. phagocytophilum* and its associated diseases by targeting similar mechanisms in both vertebrate hosts and tick vectors.

## Author contributions

AC and JF conceived the study. PA performed the experiments. AC, PA, JV, MV, and JF performed data analyses. AC and JF wrote the paper, and other co-authors made additional suggestions and approved the manuscript.

## Funding

This research was supported by the Ministerio de Economia y Competitividad (Spain) grant BFU2016-79892-P and the European Union (EU) Seventh Framework Programme (FP7) ANTIGONE project number 278976. MV was supported by the Research Plan of the University of Castilla-La Mancha (UCLM), Spain.

### Conflict of interest statement

The authors declare that the research was conducted in the absence of any commercial or financial relationships that could be construed as a potential conflict of interest.
